# Enhancing patellar positioning and tracking in robotic patello-femoral arthroplasty: a step-by-step technique

**DOI:** 10.1051/sicotj/2025040

**Published:** 2025-09-04

**Authors:** Luca Andriollo, Hannes Vermue, Carmela Pizzigallo, Jobe Shatrov, Elvire Servien, Cécile Batailler, Sébastien Lustig

**Affiliations:** 1 Orthopaedics Surgery and Sports Medicine Department, FIFA Medical Center of Excellence, Croix-Rousse Hospital, Hospices Civils de Lyon, Lyon North University Hospital 103 Grande Rue de la Croix-Rousse 69004 Lyon France; 2 Ortopedia e Traumatologia, Fondazione Poliambulanza Istituto Ospedaliero Via Bissolati 57 25124 Brescia Italy; 3 Artificial Intelligence Center, Alma Mater Europaea University Argentinierstraße 15 1040 Vienna Austria; 4 Department Orthopedic Surgery – University Hospital Ghent Corneel Heymanslaan 10 9000 Ghent Belgium; 5 Department of Anatomical, Histological, Forensic Medicine and Orthopedic Science, Sapienza University of Rome Viale Regina Elena 336 00161 Rome Italy; 6 Sydney Adventist Hospital 185 Fox Valley Road Wahroonga (Sydney) NSW 2076 Australia; 7 Sydney Orthopaedic Research Institute 66 Pacific Highway St. Leonards (Sydney) NSW 2065 Australia; 8 Univ Lyon, Claude Bernard Lyon 1 University, IFSTTAR, LBMC UMR_T9406 69622 Lyon France; 9 LIBM-EA 7424, Interuniversity Laboratory of Biology of Mobility, Claude Bernard Lyon 1 University 69100 Lyon France

**Keywords:** Patellar tracking, Functional positioning, Personalized knee arthroplasty, Robotic knee, Patello-femoral arthroplasty

## Abstract

Patellofemoral arthroplasty (PFA) is useful and effective option for treating patients with isolated patellofemoral osteoarthritis. The concept of functional positioning (FP) in PFA focuses on resurfacing the trochlea and restoring normal patellar tracking, while keeping the joint anatomy and kinematics. Even though the patellar liner cannot yet be placed with robotic assistance, robotic tools still help surgeons manage and optimize patellar tracking during surgery. This surgical technique highlights how the image-based robotic system assists the surgeon in improving patellar positioning and patellar tracking during a PFA. This technique could contribute to reduce complications, although its actual benefits remain to be validated. It may help prevent patellar instability through direct tracking assessment and reduce fracture risk by preserving more patellar bone. Accurate placement of the patellar button and evaluation of anterior offset might alleviate anterior knee pain. A tailored resection could also help protect the patellar vascular supply. Image-based planning may assist in avoiding malpositioning, potentially leading to fewer revisions.

## Introduction

Patellofemoral arthroplasty (PFA) is a useful and effective option for treating patients with isolated patellofemoral osteoarthritis (PF-OA), especially when the knee ligaments are still intact [1]. It can reduce pain, improve function, and help delay or avoid the need for a total knee replacement (TKA) [2].

In recent years, improvements in implant design have helped overcome some of the problems seen in the past, leading to better results and fewer mechanical issues [1]. Second-generation inlay implants are now shaped to better match the natural anatomy of the trochlea, which has improved the success of the procedure.

Robotic-assisted surgery has also brought major benefits. It allows for more accurate, consistent, and personalized positioning of implants based on each patient’s anatomy [3, 4]. A recent technique described by Andriollo et al. introduced the concept of functional positioning (FP) in PFA [5]. This method focuses on resurfacing the trochlea and restoring normal patellar tracking, while keeping the joint anatomy and kinematics. Even though the patellar liner cannot be placed with robotic assistance, robotic tools still help surgeons to manage and optimize patellar tracking during surgery.

**Figure 1:**
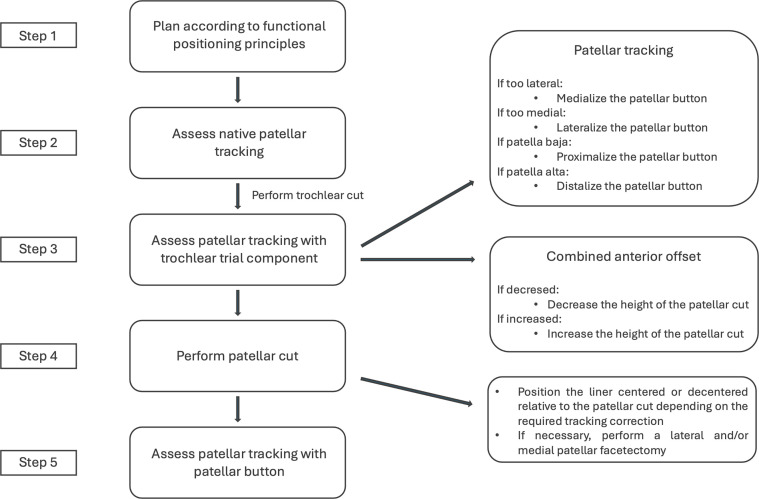
A step-by-step approach to enhance patellar positioning and tracking in image-based robotic patello-femoral arthroplasty, following the principles of functional positioning.

This surgical technique highlights how the image-based robotic system assists the surgeon in improving patellar positioning and patellar tracking during a PFA, through the description of a flow chart shown in Figure 1.

## Surgical technique

The surgical technique, demonstrated in Video 1, is an appendix to the article on FP in PFA published by Andriollo et al., which describes patient positioning, preoperative planning, the surgical approach, and pins placement.

The robot used is the Mako^®^ robotic assistance system (Stryker^®^, Mahwah, USA), with the RESTORIS MCK^®^ partial knee implant system (Stryker^®^, Mahwah, USA).

### Step 1: Plan according to functional positioning principles

After registering the bony landmarks, a trochlear mapping is performed using a dedicated tool that does not invade the cartilage. The trochlear groove is traced, and the cartilage surrounding the prosthetic implant area is mapped to accurately determine its thickness.

Using the robotic software, the intraoperative plan is finalized by adjusting the position of the trochlear implant in three planes: varus/valgus in the coronal plane, rotation in the axial plane, and flexion in the sagittal plane. This step is aimed at refining the resurfacing of the trochlea so that the implant aligns with the patient’s trochlear groove. A reference line displayed on the robotic screen guides the surgeon during this process.

### Step 2: Assess native patellar tracking

A reference point is marked at the center of the anterior surface of the patella, and the initial patellar tracking is recorded using a robotic instrument. This tracking will later help guide any necessary adjustments to improve patellar tracking during patellar preparation.

### Step 3: Assess patellar tracking with trochlear trial component

After cutting the trochlear bone using the arm-assisted burr, the trial components are placed based on the planned sizing. At this point, it is important to ensure that the resurfacing of the trochlea is accurate, making sure that no part of the implant extends beyond the edges of the cartilage. If any overhang is detected, the area must be re-cut following a re-plan.

Patellar tracking is then re-evaluated with the trial component in place. This step is crucial for assessing how the native patella tracks with the trochlea already implanted.

### Step 4: Perform patellar cut

Based on the tracking observed in Step 3, the patellar cut is planned with the goal of preserving the combined anterior offset. In fact, it is essential to ensure that the anterior offset is not increased and that patellar tracking is restored as close to neutral as possible.

To achieve this, intraoperative measurement of the patellar thickness with a caliper is required, along with knowledge of the thickness of the patellar liner.

If sagittal plane tracking with the trial components shows an increase in combined anterior offset compared to the native tracking, it is recommended to increase the depth of the patellar cut. Conversely, if the offset is reduced, it is advised to decrease the depth of the patellar cut. In all cases, the minimum residual bone thickness of the patella should be 12 mm. After the cut is made, it is recommended to use a caliper to check the accuracy of the cut in terms of thickness, as well as the uniformity of the cut, in order to avoid abnormal tilt.

When preparing the three peg holes for the patellar liner, it is important to carefully evaluate both medio-lateral and antero-posterior placement on the cut bone surface, aiming to align patellar tracking correctly with the trochlear component.

With patella baja, the patellar button should be positioned more proximally, whereas with patella alta, it should be placed more distally. If tracking is too lateral, the patellar button should be shifted medially, and if it is too medial, it should be shifted laterally. If necessary, a lateral and/or medial patellar facetectomy is performed.

### Step 5: Assess patellar tracking with patellar button

**Figure 2:**
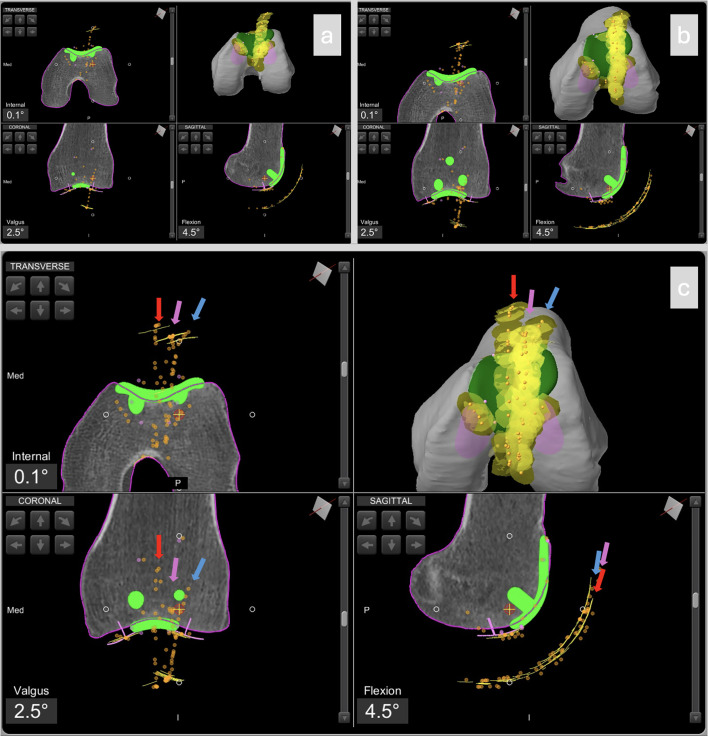
Intraoperative screenshots of patellofemoral arthroplasty planning using Mako^®^ (Stryker^®^, Mahwah, USA). The case is the same as in Video 1 (left knee). Image a) shows the initial tracking assessment (Step 2); b) patellar tracking with the trochlear trial component (Step 3); c) patellar tracking with the patellar button in place (Step 5). The blue arrow indicates the initial patellar tracking, the violet arrow shows the tracking after placement of the trochlear trial component, and the red arrow indicates the final patellar tracking.

**Figure 3:**
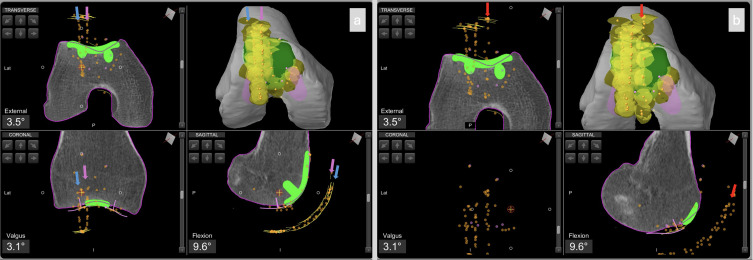
Intraoperative screenshots of patellofemoral arthroplasty planning using Mako^®^ (Stryker^®^, Mahwah, USA). The patient is the same as in Video 1 and Figure 1, but here the contralateral (right) knee is shown. Image a) shows patellar tracking with the trochlear trial component in place (Step 3); b) patellar tracking with the patellar button in place (Step 5). The blue arrow indicates the initial patellar tracking, the violet arrow shows the tracking after placement of the trochlear trial component, and the red arrow indicates the final patellar tracking.

To confirm proper management of the anterior compartment, final patellar tracking is assessed with the trial patellar liner in place (Figures 2 and 3). If tracking is satisfactory in all planes, the definitive components are cemented.

To avoid altering the offset, a precise cementation technique is required, with manual progressive compression applied to the trochlear component and a dedicated compression tool used for the patella.

## Discussion

This article describes and illustrates how enhancing patellar positioning and tracking in robotic PFA can be achieved by applying the principles of FP. In fact, FP is a concept that is becoming increasingly important to consider not only the patient’s bony anatomy, but also joint kinematics and soft tissue compliance [5–7].

The effectiveness of PFA mainly relies on selecting the right patients. When the surgical technique or the indications are not ideal, PFA tends to have higher revision rates and lower implant survival compared to TKA [8]. In the short term, many complications are linked to incorrect implant placement or poor patellar tracking, which may lead to instability, subluxation, or dislocation.

Over time, the most frequent reason for failure is the progression of tibiofemoral osteoarthritis [2]. In such cases, it may be possible to add a medial or lateral unicompartmental knee arthroplasty in a staged procedure [9, 10].

It is well recognized that anterior compartment overstuffing is linked to worse functional results [11, 12]. Robotic systems that assess patellar offset and translation relative to the trochlea during surgery support a more tailored patellar resection. This improves the accuracy of both the resection depth and the placement of the patellar component.

These steps allow surgeons to adjust the patellar cut during surgery, helping to achieve balanced patellar tracking and better alignment in the anterior compartment. This method follows the principles of FP, while maintaining native knee kinematics, especially in relation to the third space.

Recent studies suggest that greater personalization in knee arthroplasty may improve function, increase patient satisfaction, and result in more natural joint kinematics [13–15].

## Conclusions

This personalized approach to managing patellar tracking in robotic PFA using FP principles represents a promising advancement in the treatment of PF-OA.

This technique could contribute to reducing complications, although its actual benefits remain to be validated. It may help prevent patellar instability through direct tracking assessment and reduce fracture risk by preserving more patellar bone. Accurate placement of the patellar button and evaluation of anterior offset might alleviate anterior knee pain. A tailored resection could also help protect the patellar vascular supply. Image-based planning may assist in avoiding malpositioning, potentially leading to fewer revisions.

## Data Availability

The video is available as supplementary material.

## Supplementary material

*Video 1*. Surgical technique. The video is available at the following https://www.sicot-j.org/10.1051/sicotj/2025040/olm.
